# Factors influencing human papillomavirus vaccine uptake among parents and teachers of schoolgirls in Saudi Arabia: a cross-sectional study

**DOI:** 10.3389/fpubh.2024.1403634

**Published:** 2024-10-16

**Authors:** Deema I. Fallatah, Mohammad Adnan Khalil, Samar Abd ElHafeez, Salma Gouda, Huda M. Alshanbari, Maaweya Awadalla, Mamoun Ahram, Bandar Alosaimi

**Affiliations:** ^1^Department of Medical Laboratory Sciences, College of Applied Medical Sciences, Prince Sattam Bin Abdulaziz University, Al-Kharj, Saudi Arabia; ^2^Department of Basic Medical Sciences, Faculty of Medicine, Aqaba Medical Sciences University, Aqaba, Jordan; ^3^Epidemiology Department, High Institute of Public Health, Alexandria University, Alexandria, Egypt; ^4^AlHelal Specialized Hospital, Egyptian Ministry of Health, Cairo, Egypt; ^5^Department of Mathematical Sciences, College of Science, Princess Nourah Bint Abdulrahman University, Riyadh, Saudi Arabia; ^6^Research Center, King Fahad Medical City, Riyadh Second Health Cluster, Riyadh, Saudi Arabia; ^7^Department of Physiology and Biochemistry, School of Medicine, The University of Jordan, Amman, Jordan

**Keywords:** HPV vaccine, cervical cancer, human papillomavirus, factors, knowledge

## Abstract

**Introduction:**

Cervical cancer is a highly prevalent disease among women worldwide. However, the advent of a vaccine against HPV, the main cause of the disease, has prevented its spread. The acceptability of the HPV vaccine to different sectors of the Saudi community has yet to be clarified. Since parents and teachers are major influencers in the decision-making process of vaccination for HPV, this study aimed to assess the knowledge and attitudes of teachers and parents toward cervical cancer, HPV, and the HPV vaccine, and unraveled the factors that would influence recommending the vaccine.

**Methods:**

A cross sectional study was done among 927 individuals (373 teachers and 356 parents). A newly developed validated questionnaire was used to collect data on knowledge, attitude, and factors influencing cervical cancer, HPV, HPV vaccine. The relationship between different factors with knowledge and attitude were assessed using univariate and multivariate analysis.

**Results:**

Of the study participants, 94% were females, with a median (Interquartile range) age of 38(31–44) years, 12.2% were teachers, 38.7% were parents and 49.1% were parents and teachers. The majority (78.5%) were married, and 75.6% had at least one child. Among those with children, 88.6% had at least one girl, and among those with girls, 72.2% had at least one girl aged between 10 and 18 years. The total median (IQR) knowledge score was 9 [(−5)–(−26)] and the total median (IQR) attitude score was 49 (43–56). The knowledge score significantly increased by receiving postgraduate education, working in the health or education sectors, if a person knew someone diagnosed with cervical cancer, having girls in the age group of 10–18 years, reading about medical issues or having previously heard about the HPV vaccine. The attitude score significantly increased by high knowledge score and decreased if the person has previously diagnosed with cervical cancer.

**Conclusion:**

Physician’s recommendation and the amount of information on the HPV vaccine, opinions about vaccines in general, and government decrees are the main factors influencing decision on HPV vaccine Uptake. This study emphasizes the role of healthcare providers, awareness of cervical cancer, HPV and its vaccine, and social status, in favoring vaccine uptake in Saudi Arabia.

## 1 Introduction

Cervical cancer is one of the most frequent gynecological malignancies and fourth highest cause of cancer deaths among women globally (1). Cervical cancer rarely occurs in schoolgirls, however, 604,127 new cases and 341,831 deaths of middle-aged women from cervical cancer were reported in 2020 worldwide (1–3). Around 85% of mortality from cancer and more than 90% incidence of cervical cancer were reported in developing countries such as Sub-Saharan Africa (4, 5). These high figures are mainly due to the spread of the human papillomavirus (HPV), a principal cause of cervical cancer, and the lack of HPV vaccination, which is an effective preventive measure (6). Despite the significant imbalance of the cervical cancer distribution between low-income and higher-income countries, cervical cancer decreased between 1990 and 2019 due to effective preventive measures such as the Papanicolaou (Pap) test in detecting the human papillomavirus (HPV), in addition to sociocultural variables like access to healthcare, family planning, and education (7, 8). Although Saudi Arabia has a low incidence of cervical cancer, the association of HPV infection with this malignancy is comparable to the rest of the world, with HPV-16 and -18 being the two most common genotypes detected, and accounting for three-quarters of HPV infections (6).

HPV, the first known human tumor virus, is considered one of the main causes of genital, oropharyngeal, and lung malignancies. HPV was found in 50–80% of sexually active young men and women where incidence peaks in the early twenties and drops by age 55 (9). According to the World Health Organization (WHO), cervical cancers account for almost 85% of all HPV-related cancers (9, 10). HPV infection in mucosal and anogenital skin causes external general warts (EGW) that are found on the labia, clitoris, vulva, vagina, and ectocervix in women. Persistent HPV infection leads to a pre-cancer condition known as intraepithelial neoplasia or cervical intraepithelial neoplasia (CIN) based on the site of the infection. CIN can cause several complications in pregnant women such as low birth weight, pre-term birth, and death. However, screening programs and early intervention could prevent the progression of cancer (11).

Vaccination programs have significantly contributed to the prevention of HPV-associated diseases and cancer. In 2006, the first HPV vaccine was licensed to prevent infection by a mucosa tropic sexually transmitted infectious agent without specific induction of mucosal immunity, by targeting HPV-16 and HPV-18 types that cause the majority of HPV-attributable cancers (12). HPV vaccines are virus-like particle vaccines (VLPs) from L1 major capsid protein, that are introduced in 2–3 doses to girls and boys aged 9–14 years. Many observational studies evaluated the effectiveness and the appropriate number of doses; an initial report showed that three doses were the most effective, especially for people who received the vaccine at a younger age. However, over time, more studies have shown greater effectiveness with fewer doses (13). Recently, a clinical trial compared single-dose and three doses vaccination, showing high efficacy and long-term protection with a single dose of HPV vaccine (14).

By June 2020, 107 (55%) of the 194 WHO member states reported the implementation of HPV vaccination programs worldwide in their national immunization schedules. The HPV program’s average coverage and performance is significantly lower than that of childhood immunizations, and collaborative efforts are required to achieve the 2030 WHO elimination targets (9, 10). Many studies have been undertaken worldwide to evaluate HPV awareness and vaccination acceptability among parents. European studies reported that HPV-related knowledge was poor, especially in addressing diseases in men. Despite the limited degree of awareness, participants’ attitudes were generally encouraging (15–17). Many factors contributed to parents’ acceptance of the vaccination, including demographic characteristics such as female gender, younger parent age, female adolescent gender, higher household income, and past childhood vaccination history. In addition, the belief in vaccine efficacy in preventing cervical cancer, awareness of HPV, susceptibility to HPV infection, doctors’ awareness, desire to conform to social norms, and perception of disease severity, have all been reported to influence vaccine acceptance (18–21). However, the most common impediments to HPV vaccination uptake are concerns about the vaccine’s safety (17), beliefs about its impact on sexual behavior, low perceived vulnerability to HPV infection, and uncertainties about its efficiency (17, 22, 23). Vaccine hesitancy had increased worldwide before the COVID-19 pandemic and increased further during it (24). Cultural differences and religious beliefs play a vital role in vaccine acceptance. Religion has a positive correlation with susceptibility and the benefits of vaccination, but the significance of religion regarding beliefs about the HPV vaccine varies worldwide (21, 25–28).

In Saudi Arabia, the HPV vaccination program was only established in 2020. There is, therefore, not enough data collected on population acceptance of the vaccine (29–31). Since HPV vaccination program in Saudi is conducted at schools of young girls, the success of the program largely depends on the teachers’ and parents’ awareness and decisions. Therefore, evaluating guardians’ and teachers’ knowledge, attitude, and perception of HPV vaccination is crucial to gaging the extent of health education and level of vaccine hesitancy. In this study, we aim to assess guardians’ and teachers’ knowledge and attitudes toward the HPV vaccine and determine factors that influence vaccine uptake among schoolgirls in Saudi Arabia.

## 2 Materials and methods

### 2.1 Study design and sampling technique

This is a cross-sectional study that was conducted in the middle region of Riyadh City in Saudi Arabia. Riyadh City is the capital of Saudi Arabia and is divided into 5 regions; the middle region is a highly populated and dense area with a population density of 22.5 people per square kilometer (32).

A multi-stage sampling technique was used to select districts and schools for the study. The middle region consists of five districts, from which three districts—Alsulimaniah, Alolaya, and Almalaz—were randomly selected. The total number of girls-only schools in the middle region is approximately 80, with about 16 schools per district. In the three selected districts, there are 50 girls-only schools. The variability within clusters is nearly equivalent to that of independent sampling, making our approach comparable to simple random sampling. Since the schools selected from the districts are considered socially homogeneous, this supports the assumption of minimal clustering effects. From these, 15 schools were randomly selected to be included in the study, with the number of selected schools proportionally allocated according to the number of schools in each district. Across these 15 schools, the approximate total number of students and teachers is 4,300 and 500, respectively.

### 2.2 Study population and sample size

#### 2.2.1 Sample size for assessing the psychometric properties of the questionnaire

Based on the sample size recommendation for validating a questionnaire (33), we included 100 participants to assess the psychometric properties of the questionnaire (50 teachers and 50 parents). The total sample size was 729 respondents; including 373 teachers and 356 parents.

#### 2.2.2 Sample size of teachers

Given the lack of prior research assessing the knowledge of teachers about HPV and its vaccine in Saudi Arabia, we estimated our sample size based on an assumption that 42% of female high school teachers in Saudi Arabia have good knowledge of cervical cancer screening (29). Using OpenEPI, we calculated the minimum required sample size with a margin of error of 5.0%, an alpha error of 0.05, and a non-response rate of 10%. This resulted in the required sample size of 237 teachers. However, to enhance the robustness of our study and account for potential variability, we ultimately included 373 teachers, recruited proportionally from each of the selected schools.

#### 2.2.3 Sample size of parents

The sample size for parents was calculated assuming that 32.9% of parents have good knowledge of HPV and the HPV vaccine in Saudi Arabia (30)., Using OpenEPI, we calculated the minimum required sample size with a margin of error of 5.0%, an alpha error of 0.05, and a non-response rate of 10%. The minimum required sample size was calculated to be 347 parents, but we aimed for a higher number to account for potential variability (34). Ultimately, we received 356 responses from parents, who were invited to participate by their children.”

### 2.3 Study phases

Phase 1: Development and validation of the questionnaire to assess the knowledge and attitude of teachers and parents on cervical cancer, HPV, and the HPV vaccine. A group from the research team, experienced in questionnaire development and validation, held five meetings to identify the constructs and develop items under each construct.

Phase 2: Expert evaluation. An expert panel, consisting of five investigators (one methodologist, one healthcare professional, one gynecologist, and two language professionals) assessed the questionnaire for clarity and determined whether the identified items covered the defined constructs to ensure face and content validity.

Phase 3: Pilot testing and cognitive interviews. A pilot test of the pre-final questionnaire was carried out through cognitive interviews of 20 intended respondents (10 teachers and 10 parents) to evaluate their understanding, and the readability, syntax, wording, cultural appropriateness and clarity of the items.

Phase 4: Testing the questionnaire’s psychometric properties. A sample of 100 teachers and parents (50 for each group) were identified to test the reliability and validity of the pre-final version of the questionnaire.

### 2.4 Data collection tool

The final version of the questionnaire was produced in Arabic and verified by the authors who are all native Arabic speakers. The English translation was used for the manuscript and verified by three of the authors (ASA, MAK, and MA2). The questionnaire items were divided into four sections. The first section focused on basic socio-demographic data. It also included questions about the participants’ familiarity with cervical cancer and sources of information on the HPV vaccine. The second section consisted of 16 items with a choice of answers (‘Correct’, ‘False’, ‘Do not know’) to evaluate the respondents’ knowledge of cervical cancer, HPV, and the HPV vaccine. The third section comprised 15 items, each having five response options based on a Likert scale ranging from ‘Strongly disagree’ to ‘Strongly agree’ to assess respondents’ attitudes toward cervical cancer, HPV, and the HPV vaccine. Finally, the fourth section was composed of 13 items to identify the factors that affected the decision of HPV vaccination uptake on a Likert scale ranging from ‘Strongly positive’ to ‘strongly negative.’

The knowledge items were scored as follows: −1 for ‘Do not know’, 0 for ‘False’, and 1 for ‘Correct’. The correct answers were weighted according to the level of difficulty of each item. The maximum score on the knowledge section was 51, indicating good knowledge. The attitude questions in section three were scored using a five-point Likert scale as follows: 1 point for ‘Strongly disagree’, 2 points for ‘Disagree’, 3 points for ‘Not sure’, 4 points for ‘Agree’, and 5 points for ‘Strongly agree’. The maximum score on the attitude section was 60, indicating a very positive attitude.

### 2.5 Statistical analysis

#### 2.5.1 Psychometric evaluation of the questionnaire

Content validity was assessed by an expert panel of five investigators with knowledge and expertise in instrument development. Content clarity was determined for all items. Convergent validity was assessed by calculating item-total correlations for each construct of the questionnaire. Divergent validity was assessed by measuring the correlation between total scores for each construct.

The Cronbach’s *α* coefficient was used to assess the internal consistency of the questionnaire.

#### 2.5.2 Data management

Data distribution of the final version of the questionnaire was checked using visual identification of a normal distribution by QQ plot. Quantitative variables were summarized as median [interquartile range (IQR)] for non-normally distributed data. Qualitative variables were presented as percentages and frequencies.

Bivariate analysis was carried out using Mann–Whitney and Kruskal-Wallis tests to compare knowledge and attitude scores versus the baseline characteristics of the study population. Correlation analysis was conducted using Spearman’s rho test. Two multiple linear regression models were built to identify the predictors of knowledge and attitude scores. All potential confounders were included in the analysis. The final models included the following variables: age group, education, marital status, job category, group category (parents or teachers), reading about medical issues, having girls in the age group from 10 to 18 years, participants’ knowledge about someone who has been diagnosed with cervical cancer, participants’ self-diagnosis of cervical cancer, and participants’ hearing about cervical cancer. In addition, a knowledge score was added to the multiple linear regression model for identifying predictors of attitude. The Statistical Package for the Social Sciences (SPSS), version 20.0, for Windows was used. The tests were two-tailed, and *p* values <0.05 were considered to indicate statistical significance.

## 3 Results

### 3.1 Piloting and validation

The mean age of the 100 study participants included in this phase was 39.9 ± 6.6 years; 89% were females and 79.1% were married. Analyses of convergent validity revealed that all items in all sections significantly correlated with the total score (*p* < 0.001). Analyses of divergent validity revealed that the total scores of ‘knowledge of HPV, HPV vaccine, and cervical cancer’ significantly correlated with ‘attitude toward HPV, HPV vaccine and cervical cancer’ (*r* = 0.39, *p* < 0.001).

Reliability analyses revealed acceptable Cronbach’s *α* scores for all sections. The score for the ‘knowledge of HPV, HPV vaccine, and cervical cancer’ section had a Cronbach’s α of 0.93, and the score for the attitude toward HPV, HPV vaccine, and cervical cancer had Cronbach’s alpha of 0.92. The final section on the factors affecting HPV vaccine decision had a Cronbach’s alpha of 0.93, after removing two individual items from the factors affecting the participant’s decision to take the HPV vaccine that had a Cronbach’s alpha less than 0.7. The two factors were ‘the vaccine administration requires injection with needles’ and ‘the global prevalence of cervical cancer’.

### 3.2 Characteristics of the study population

In this study, 729 participants answered the questionnaire, with a median (IQR) age of 38 (31–44) years; 94% were females. Among them, 12.2% were teachers, 38.7% were parents and 49.1% were both parents and teachers. 74.5% held a bachelor’s degree and 46.5% were employed in the government sector, with 55.1% working in the education field. The majority (78.5%) were married, and 75.6% had at least one child. Among those with children, 88.6% had at least one girl, and among those with girls, 72.2% had at least one girl aged between 10 and 18 years. Almost two-thirds (64%) indicated that they read about medical issues (Table 1).

**Table 1:** Baseline characteristics of the study participants (*n* = 729).

Demographic variable	Frequency	%
**Age**	Median (IQR) = 38 (31–44)	
Gender		
Male	44	6.0
Female	685	94.0
Groups		
Parents	282	38.7
Teachers	89	12.2
Parents and teachers	358	49.1
Educational level		
Diploma	124	17.0
Bachelor	543	74.5
Master	41	5.6
Doctorate	21	2.9
Employment sector		
Unemployed	174	23.9
Private or international	216	29.6
Government	339	46.5
Job field		
Unemployed	178	24.4
Interdisciplinary	76	10.4
Education	415	56.9
Health	60	8.2
Marital status		
Never married	157	21.5
Married	535	78.5
Number of children		
None	177	24.4
One	57	7.8
Two	108	14.8
Three	117	16.0
Four or more	270	37.0
Number of girls (*n* = 552)		
None	63	11.4
One	172	31.2
Two	154	27.9
Three	102	18.5
Four or more	61	11.1
Number of girls aged 10 to 18 (*n* = 493)		
None	136	27.8
One	172	35.2
Two	106	21.7
Three	50	10.2
Four or more	25	5.1
Reading about medical issues		
Never	31	4.3
Yes, a little	231	31.7
Yes, somewhat	332	45.5
Yes, a lot	135	18.5
Familiarity with cervical cancer		
Participant knows someone who has been diagnosed with cervical cancer	199	27.3
Participant has been diagnosed with Cervical cancer (*n* = 685)	10	1.5
Participants heard about HPV vaccine	596	82

**Figure 1:**
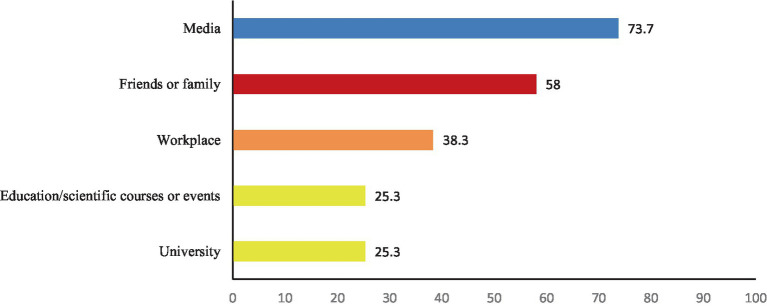
Sources of information on the HPV vaccine among the study participants.

27.3% of participants reported knowing someone diagnosed with cervical cancer, while only 1.5% of female participants indicated a personal diagnosis of cervical cancer. Most of the study population (82%) reported being aware of the HPV vaccine. The main source of their knowledge was the media, indicated by 73.7%, followed by family or friends (58%) and the workplace (38.3%). Educational and scientific events at the university were the least sources of information, both of which were selected by 25.3% of respondents (Table 1; Figure 1).

### 3.3 Knowledge of cervical cancer, HPV, and the HPV vaccine

Participants were tested for their knowledge regarding cervical cancer and the HPV vaccine by showing them several statements and asking them to indicate if they knew if the statements were true or false, or if they had no knowledge of their veracity. The minimal score of knowledge was −13 and the maximum score was 51. The total median (IQR) score of knowledge score was 9 [(−5)–(−26)], which reflects poor knowledge of many items. More than half of the study population failed to select the correct answers to the following statements: ‘Symptoms of cervical cancer include pain during intercourse and vaginal bleeding after intercourse’; ‘There is a PCR test for HPV’; ‘HPV infection has obvious symptoms in the female genital system,’; ‘Sexual contact is the main mode of HPV transmission,’; and “There is currently a treatment to cure HPV infection’. Additionally, more than 60% did not know that ‘HPV is the main cause of all types of cervical cancer’; ‘men, like women, can get infected with HPV’; ‘there are different strains of HPV’; ‘there are five stages for the progression of cervical cancer’; and ‘there are different strains of HPV’ (Table 2).

**Table 2:** Knowledge of cervical cancer, HPV, and the HPV vaccine (*n* = 729).

	Do not know	False	True
HPV is the main cause of all types of cervical cancer	461(63.2)	52(7.1)	216(29.6)
Symptoms of cervical cancer include pain during intercourse and vaginal bleeding after intercourse	389(53.4)	41(5.6)	299(41)
A cervical swab diagnoses cervical cancer	238(32.6)	34(4.7)	457(62.7)
When diagnosed early enough, cervical cancer is treatable and curable	212(29.1)	34(4.7)	483(66.3)
There are five stages for the progression of cervical cancer	493(67.6)	32(4.4)	204(28)
HPV infection caused obvious symptoms in the female genital system	405(55.6)	45(6.2)	279(38.3)
There is a PCR test for HPV	393(53.9)	48(6.6)	288(39.5)
There is currently a treatment to cure HPV infection	451(61.9)	39(5.3)	239(32.8)
Sexual contact is the main mode of HPV transmission	423(58)	81(11.1)	225(30.9)
Men, like women, can get infected with HPV	470(64.5)	100(13.7)	159(21.8)
There are different strains of HPV	509(69.8)	27(3.7)	193(26.5)
Getting vaccinated with the HPV vaccine is recommended by the Saudi MOH	277(38)	23(3.2)	429(58.8)
HPV vaccine must be given to girls aged 9 to 13 years old	0(0)	431(59.1)	298(40.9)
HPV vaccine prevents 70% of cervical cancer cases	0(0)	408(56)	321(44)
HPV vaccine is given in two doses separated by 6–12 months	0(0)	519(71.2)	210(28.8)
HPV is given intramuscularly	239(32.8)	240(32.9)	250(34.3)
Total knowledge score [median (IQR)] Minimum-maximum	9((−5)-26) (−13)-51		

Conversely, more than 50% of the participants provided an accurate response to the statement ‘Getting vaccinated with the HPV vaccine is recommended by the Saudi MOH’, whereas 62.7% provided the correct answer to ‘A cervical swab diagnoses cervical cancer, and 66.3% responded correctly to ‘When diagnosed early enough, cervical cancer is treatable and curable’ (Table 2).

### 3.4 Attitudes toward cervical cancer, HPV, and HPV vaccine

We then assessed the attitude of respondents toward cervical cancer, HPV, and the HPV vaccine. The total median (IQR) score was 49 (43–56). As the possible range was 14–70, the score reflects a favorable attitude. Over half of the study participants agreed on several items such as, ‘I must encourage my students/daughters to take the HPV vaccine’; ‘My recommendation in favor of getting the HPV vaccine does not necessarily mean that I agree to give it to my family’; ‘I will not recommend the HPV vaccine if it has adverse effects’; ‘I will not recommend the HPV vaccine if it becomes clear that it will lead to community disintegration and the introduction of concepts of sexual liberation’; and ‘Religious jurisdiction has a great effect on encouraging vaccination with the HPV vaccine.’ Additionally, more than 60% agreed that ‘I recommend getting vaccinated with the HPV vaccine to obtain essential protection against cervical cancer’; ‘medical recommendations from experts have a great effect on encouraging vaccination with the HPV vaccine’; and, ‘awareness campaigns about the importance of the HPV vaccine will encourage its administration’ Conversely, more than 40% of the study population were unsure about the following statements: ‘I think that the HPV vaccine is effective’ and ‘I think that the HPV vaccine is safe’ (Table 3).

**Table 3:** Attitudes toward cervical cancer, HPV, and the HPV vaccine (*n* = 729).

	Strongly disagree	Disagree	Not sure	Agree	Strongly agree
I think that the HPV vaccine is effective	16(2.2)	39(5.3)	342(46.9)	168(23)	164(22.5)
I think that the HPV vaccine is safe	16(2.2)	37(5.1)	370(50.8)	160(21.9)	146(20)
I must encourage my students/daughters to take the HPV vaccine	18(2.5)	56(7.7)	252(34.6)	190(26.1)	213(29.2)
Awareness campaigns about the importance of the HPV vaccine will encourage its administration	14(1.9)	45(6.2)	162(22.2)	184(25.2)	324(44.4)
Girls must be enabled to take the HPV vaccine without their parent’s permission	82(11.2)	177(24.3)	228(31.3)	102(14)	140(19.2)
‘Sex education’ must be included in the school curriculum	43(5.9)	106(14.5)	221(30.3)	181(24.8)	178(24.4)
Religious jurisdiction has a great effect on encouraging vaccination with the HPV vaccine	22(3)	58(8)	239(32.8)	190(26.1)	220(30.2)
Medical recommendations from experts have a great effect on encouraging vaccination with the HPV vaccine	18(2.5)	36(4.9)	172(23.6)	199(27.3)	304(41.7)
My experience with the COVID-19 vaccine encourages me to advocate for vaccination with the HPV vaccine*	43(5.9)	101(13.9)	222(30.5)	174(23.9)	184(25.2)
I recommend getting vaccinated with HPV vaccine to obtain essential protection against cervical cancer*	20(2.7)	50(6.9)	212(29.1)	212(29.1)	229(31.4)
My recommendation in favor of getting HPV vaccine does not necessarily mean that I agree to give it to my family*	35(4.8)	78(10.7)	226(31)	206(28.3)	175(24)
I will not recommend the HPV vaccine if religious scholars advised against it*	35(4.8)	99(13.6)	254(34.8)	164(22.5)	169(23.2)
I will not recommend the HPV vaccine if it has adverse effects*	31(4.3)	80(11)	219(30)	163(22.4)	225(30.9)
I will not recommend the HPV vaccine if it becomes clear that it will lead to community disintegration and the introduction of the concepts of sexual liberation*	44(6)	80(11)	213(29.2)	154(21.1)	233(32)
I must avoid talking about sexual education with my students/daughters	73(10)	243(33.3)	217(29.8)	113(15.5)	83(11.4)
Total attitude score (median (IQR)) Minimum-maximum	49(43–56) (14–70)				

*Missing cases in these questions range from 0.6 to 1.5%.

### 3.5 Associations between the participants’ baseline characteristics and the knowledge and attitude scores for cervical cancer, HPV, and/or the HPV vaccine

The knowledge score exhibited notable differences across various baseline characteristics. For instance, overall knowledge and higher educational status and knowledge of medical issues and the HPV vaccine were significantly associated with higher median knowledge scores of cervical cancer, HPV, and the HPV vaccine. In addition, those who were unemployed [6 (−11)–21] had a significantly lower score regardless of the sector they worked in, although those engaged in the health sector demonstrated the highest score [21 (7.5–35)]. Furthermore, participants with at least one child had a significantly higher score [6.5 (−7–24)] and those with girls aged between 10 and 18 years had higher scores than those without either [4 (−12–25.75)]. Participants who knew someone who was previously diagnosed with cancer of the cervix also had a significantly higher score compared to those who did not know someone who had been diagnosed [13 (−1–25)] vs. 3 (−10.5–23.5; Table 4).

**Table 4:** Associations between participants’ baseline characteristics and knowledge and attitudes scores regarding cervical cancer, HPV, and the HPV Vaccine by their demographic characteristics.

	Knowledge			Attitudes		
	Median (IQR)	*p*-value	Spearman’s rho (*p*)	Median (IQR)	*p*-value	Spearman’s rho (*p*)
Group						
Parents	9.5((−6.3)-26)	0.69	0.0(0.99)	49(43–56)	0.73	0.02(0.58)
Teachers	9((−8.5)-26.5)			50(42–57)		
Parents and teachers	9.5((−4.0)-25)			49(43.8–56)		
Gender						
Male	6 (−7–23.75)	0.10	−0.06(0.10)	49 (43–56)	0.42	−0.03(0.42)
Female	−7 (−13–13)			50.8 (42.75–57.25)		
Age group						
Fewer than 30 years	5.5 (−12 – 25.75)	0.57	0.02(0.57)	48.5 (43–57)	0.55	−0.02(0.55)
30 years or more	6 (−7–22.5)			49 (43–56)		
Marital status						
Never married	4.5 (−11.75–23.75)	0.09	0.06(0.09)	49.5 (43.25–57)	0.61	−0.02(0.61)
Married	6 (−7.25–24)			49 (43–55)		
Job description						
Unemployed	6 (−11)–21	<0.001	0.14(<0.001)	47 (42–55)	0.03	0.09(0.01)
Health	21 (7.5–35)			50 (46–57)		
Other	11 (−2.75–27.5)			51 (46–56)		
Education	9 (−6–25.2)			49 (43–56)		
Years of experience						
Fewer than 25 years	6 (−9–24)	0.73	−0.14(0.73)	49 (43–56)	0.36	0.04(0.36)
25 years or more	4 (−12–17)			55 (39–62)		
Education						
Undergraduate	5 (−9–24)	0.01	0.09(0.01)	48.5 (43–56)	0.62	0.02(0.36)
Postgraduate	13 (−3.25–32.25)			51 (44.75–56.25)		
Reading about medical issues						
No	−10 (−12.25)–(−6.75)	0.02	0.09(0.02)	44.5 (41.25–57.25)	0.11	0.06(0.11)
Yes	6 (−7–24.75)			49 (43.25–56)		
Number of children						
None	4 (−12 – 25.75)	0.04	0.08(0.04)	49 (43–57)	0.97	−0.002(0.97)
At least one	6.5 (−7–24)			49 (43–55.25)		
Number of girls aged 10 to 18						
None	4 (−12–24)	0.01	0.09(0.01)	48 (43–57)	0.47	−0.03(0.47)
At least one	8 (−5–26)			49 (44–55)		
The participant has been diagnosed with cervical cancer						
No	3 (−10.5 – 23.5)	0.17	0.05(0.17)	48 (43–56)	0.28	−0.04(0.28)
Yes	13 (−1 – 25)			49 (38.75–52.25)		
The participant knows someone who has been diagnosed with cervical cancer						
No	3 (−10.5 – 23.5)	<0.001	0.14(<0.001)	48 (43–56)	<0.001	0.1(0.001)
Yes	13 (−1 – 25)			50 (43–57)		
Participants heard about HPV vaccine						
I never heard of it	4 (−9–20.5)	0.01	0.09(0.01)	48 (42–54.5)	0.048	0.07(0.048)
I heard about it from at least one source	11 (−4–26)			50 (43–56)		

As for attitudes toward the HPV vaccine, the median (IQR) attitude scores exhibited notable differences across various baseline characteristics in the study population. Specifically, participants employed in the health [50 (46–57)], education [49 (43–56)], or other sectors [51 (46–56)] displayed significantly higher scores compared to those who were unemployed [47 (42–55)]. Moreover, favorable attitudes were also significantly measured in participants who knew someone diagnosed with cervical cancer or if the participants had heard about the HPV vaccine (Table 4).

### 3.6 Predictors of knowledge and attitude for cervical cancer, HPV, and/or HPV vaccine

We then analyzed predictors of knowledge score of cervical cancer, HPV, the HPV vaccine, and attitudes toward it. Table 5 indicates that a significant increase in the knowledge score could be predicted by various factors, such as receiving postgraduate education (B: 5.96, 95% CI: 0.83–11.08), working in the health sector (B: 11.43, 95% CI: 5.74–17.13), and working in the education sector (B: 7.11, 95% CI: 0.80–13.42). Additionally, the knowledge score could be positively predicted if a person knew someone diagnosed with cervical cancer (B: 4.58, 95% CI: 1.45–7.70), having girls in the age group of 10–18 years (B: 3.87, 95% CI: 0.58–7.17), reading about medical issues (B:7.16, 95%CI:0.32–13.99) or having previously heard about the HPV vaccine (B: 5.56, 95% CI: 1.98–9.15).

**Table 5:** Predictors of knowledge and attitudes toward cervical cancer, HPV, and the HPV vaccine.

Variables	Knowledge		Attitude	
	Unstandardized B (95% CI)	*p*-value	Unstandardized B (95% CI)	*p*-value
Age group (30 years and above vs. less than 30)	−3.79(−8.16–0.59)	0.09	−0.09(−2.06–1.84)	0.93
Gender (male vs. female)	−4.61(−10.62–1.41)	0.13	1.02(−1.70–3.74)	0.46
Group				
Parents vs. parents and teachers	4.16(−1.98–10.30)	0.18	−0.04(−2.81–2.74)	0.98
Teachers’ vs. parents and teachers	2.73(−2.67–8.13)	0.32	−0.003(−2.44–2.44)	0.99
Marital status (ever married vs. never married)	3.88(−1.07–8.83)	0.12	−0.91(−3.14–1.33)	0.43
Education (postgraduate vs. undergraduate)	5.96(0.83–11.08)	0.02	−1.17(−3.49–1.15)	0.32
Job sectors				
education sector vs. non-employed	7.11(0.80–13.42)	0.03	0.89(−1.96–3.75)	0.54
health sector vs. non-employed	11.43(5.74–17.13)	<0.001	0.04(−2.56–2.64)	0.98
other sectors vs. non-employed	1.96(−2.76–6.67)	0.42	0.41(−1.72–2.54)	0.70
Reading about medical issues (Yes vs. No)	7.16(0.32–13.99)	0.04	0.31(−2.78–3.41)	0.84
The participant has been diagnosed with cervical cancer (Yes vs. No)	2.73(−9.87–15.33)	0.67	−6.12(−11.80-(−0.043))	0.04
The participant knows someone who has been diagnosed with cervical cancer (Yes vs. No)	4.58(1.45–7.70)	0.004	1.22(−0.20–2.64)	0.09
Number of girls aged 10 to 18 (At least one vs. none)	3.87(0.58–7.17)	0.02	−7.8(−2.28–0.71)	0.30
Participants heard about HPV vaccine (From at least one source vs. never heard)	5.56(1.98–9.15)	0.002	0.36(−1.27–1.99)	0.67
Knowledge score			0.24(0.21–0.27)	<0.001

An attitude score significantly decreased if a participant was previously diagnosed with cervical cancer [B: −6.12, 95% CI: −11.80−(−0.43)]. On the other hand, higher levels of knowledge of cervical cancer, HPV, and the HPV vaccine positively increased attitude score (B: 0.24, 95% CI: 0.21–0.27; Table 5).

### 3.7 Factors affecting the participant’s decisions toward HPV vaccine

**Figure 2:**
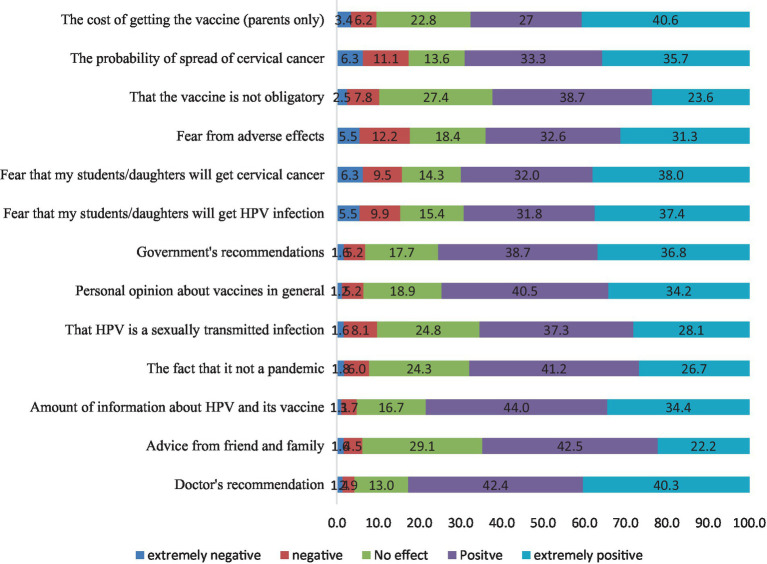
Factors affecting the participants’ decisions toward accepting the HPV vaccine.

Finally, we investigated the factors that would influence a person’s decision to take or recommend the HPV vaccine. As shown in Figure 2, the majority of the study population (82.7%) was positively influenced by the physician’s recommendation as the most crucial factor for HPV intake. Nearly three-quarters of participants mentioned that the amount of information on the HPV vaccine, opinions about vaccines in general, and government decrees could positively impact their decision. Other substantial positive factors that were found to influence the decision of 60–70% of the study population included the fear of being affected by cervical cancer, HPV or adverse events; the fact that cervical cancer and HPV infection are not ‘endemic’, the cost and the fact that the vaccine was not compulsory, and receiving ‘advice from friends or family’.

## 4 Discussion

HPV is a common sexually transmitted infection that can cause a range of health issues, including cervical cancer, as well as other types of cancers (35, 36). The HPV vaccine is a preventive measure that can eradicate several types of HPV (37) and, hence, is highly effective in reducing the risk of HPV infection and related diseases (37, 38). We investigated the knowledge of a critical sector of Saudi society in decision-making about accepting the HPV vaccination, namely parents and teachers.

Survey respondents had good knowledge regarding the recommendation of the Saudi Ministry of Health (MOH) regarding HPV vaccination, the effectiveness of a cervical swab in diagnosing cervical cancer, and the importance of early diagnosis. However, the results also revealed an alarming lack of knowledge regarding various aspects of cervical cancer, the HPV infection, and the HPV vaccine, suggesting a lack of awareness and understanding. For instance, respondents were not aware of the symptoms of cervical cancer, the main mode of HPV transmission, its causative association with cervical cancer, the existence of molecular tests for HPV, or even the availability of treatment for HPV infection.

The results of this study also illustrated a range of attitudes and opinions regarding the HPV vaccine. It is positive to note that over half of the participants agreed on several items, such as the importance of encouraging students/daughters to take the HPV vaccine and the potential impact of medical recommendations and awareness campaigns on vaccine administration. These findings suggest that there is a willingness to promote the vaccine and recognize the value of vaccination in protecting against cervical cancer.

The results showed notable differences in knowledge levels, attitudinal scores and predictors. They can be summarized into three subsets. The first is the nature of the profession and exposure to information. Understandably, employed individuals, particularly in the health sector, or those holding a postgraduate degree, had significantly better knowledge compared to others (39–44). Social status is another important factor. Specifically, participants with at least one child and, particularly those with girls aged between 10 and 18 years, had a substantially better knowledge score than those without children or girls in that age group. This could be because individuals with children may be more concerned about their family’s health and therefore seek out information about cervical cancer and HPV and the importance of HPV vaccination (45). Another influential aspect was personal experience or interactions with individuals affected by cervical cancer, which increased knowledge and awareness of the disease. Interestingly, although being informed about medical issues also had a positive impact regarding knowledge, it was neither associated with positive attitudes toward the HPV vaccine nor was it a predictor of either knowledge or attitude. Collectively, better knowledge about cervical cancer, HPV, and/or the HPV vaccine and concern about family well-being was associated with a more favorable attitude.

A study conducted among 117 undergraduate female students found that 44% of the participants were willing to be vaccinated against HPV. The young undergraduates’ intentions to receive the HPV vaccine were associated with high levels of knowledge about risk factors for cervical cancer and their perceptions that infected women are responsible for their own infection of HPV (46). A similar study among health professionals reported that 77.2% of respondents were willing to be vaccinated and recommend HPV vaccination to their family members (39). the latter study also found that the most common reason for not being vaccinated against HPV was the lack of awareness. Higher willingness (92.8%) to get vaccinated or recommended that their relatives and friends get vaccinated was reported (40). The willingness to receive HPV vaccination in our study was moderate, despite the low knowledge level of cervical cancer prevention and treatment among college students. Interestingly, two recent studies have reported a similar proportion of acceptance among parents to vaccinate their daughters (41, 42), in contrast to a study reporting less than 50% of survey participants supporting the vaccination of their daughters (43) and another revealing overwhelming refusal (94.4%) of vaccinating their daughters (44). Lack of knowledge of HPV and the HPV vaccine was a main reason for parents not accepting vaccinating their daughters among these studies. It is also worth noting that a significant portion of the parents in many of the aforementioned studies did not merely reject the vaccine, but expressed uncertainty or did not strongly agree with statements related to the effectiveness and safety of the HPV vaccine. This again highlights the need for further education and awareness campaigns to address concerns and misconceptions surrounding the HPV vaccine (47–50).

The prevalence of low knowledge is not limited to Saudi Arabians, but also to Arab women in general (51), as well as globally. For example, in one study, only 15.7% of Chinese women had a good understanding of cervical cancer (52). In the same study, the level of education was positively associated with awareness of the HPV vaccine (52). The same is true in our study where higher education and increasing knowledge were associated with increased awareness.

We also explored the key factors affecting participants’ decisions to take or recommend the HPV vaccine. The majority of the study participants (82.7%) considered the physician’s recommendation as the most crucial factor influencing their decision to take the HPV vaccine. This finding highlights the significant influence of healthcare professionals and their opinions in promoting vaccine uptake (53, 54). A positive recommendation from a trusted physician or health worker may provide reassurance and increase confidence in the vaccine’s safety and effectiveness. Another important positive factor was the information available on the HPV vaccine. Access to accurate and comprehensive information is essential for individuals to make informed choices (55). Educating the public about the benefits of the vaccine, its safety profile, and the diseases it prevents could contribute to increased acceptance and uptake. Participants who received recommendations from healthcare professionals were more likely to have positive attitudes toward the vaccine and to consider vaccinating themselves or their children. This emphasizes the importance of healthcare providers in promoting the HPV vaccination and underscores the need for comprehensive training programs to equip them with the knowledge and skills to address vaccine-related concerns and provide accurate information.

This study revealed that participants considered government decisions to be a positive influence on their decision to receive the HPV vaccine (56, 57). There appears to be a high level of public trust in the recommendations mandated by the government. Public health policies and recommendations play a significant role in shaping public perception and acceptance of vaccines (58). Clear and supportive governmental guidelines enhance public trust and encourage individuals to prioritize vaccination (56).

Participants’ opinions about vaccines in general were also found to influence their decision to receive the HPV vaccine. Positive views on vaccines, such as recognizing their role in disease prevention and public health, influenced individuals to be more receptive to the HPV vaccine (47). There were other influential factors such as misconceptions regarding the vaccine in associating it with increased infection with HPV, infection with cervical cancer, or suffering from adverse side-effects.

### 4.1 Limitations of the study

It is important to consider the limitations and potential biases of our study when interpreting the findings. The study relied on self-reported data, which can be subject to recall bias or social desirability bias. Participants may have provided answers that they believed were expected or socially acceptable, which could impact the accuracy of the results. The occupational distribution of the participants revealed that half were teachers, indicating that a significant proportion of the sample worked in the field of education. Furthermore, a majority of the participants held a bachelor’s degree, highlighting a relatively high level of education within the study population. These factors provide a more nuanced interpretation of the study’s findings and identify areas for further research. Long-term follow-up studies are also needed to assess the durability and sustainability of changes in knowledge and attitudes. They would provide insights into the long-term impact of interventions and identify potential attenuation or decay of effect over time.

## 5 Conclusion and recommendations

This study provides valuable insights into the knowledge, attitudes, and perceptions toward HPV vaccination in Saudi Arabia. While the findings indicate a moderate level of knowledge and positive attitudes toward vaccination, there are still significant knowledge gaps and cultural factors that need to be addressed to enhance vaccine acceptance. Public health interventions should focus on improving awareness of cervical cancer, and HPV, and its vaccine to change negative attitudes toward the HPV vaccine and the decision to take it or recommend it, address misconceptions, and engage healthcare providers and community leaders to promote vaccination and reduce the burden of HPV-related diseases in Saudi Arabia. Interventions should take into account negative attitudes or vaccine hesitancy that may act as a barrier to uptake. This highlights the role of healthcare providers in influencing HPV vaccination decisions. It is probable that the controversies surrounding vaccines, particularly during the COVID-19 pandemic, influenced respondents (48). The decision to receive the HPV vaccine is, however, influenced by a variety of factors, including individual, interpersonal, and societal elements. When developing strategies to increase acceptance and uptake of the vaccine, healthcare professionals, policymakers and public health authorities should consider these factors. We recommend that cultural considerations should be considered when designing education campaigns for the Saudi community (49). Additionally, it is important to align the campaign with governmental decrees, and religious and social values of the local community, ensuring that the information does not contradict religious beliefs or cultural practices (50).

## Data Availability

The original contributions presented in the study are included in the article/Supplementary material, further inquiries can be directed to the corresponding author.
