# Supplementary material for: Factors influencing human papillomavirus vaccine uptake among parents and teachers of schoolgirls in Saudi Arabia: a cross-sectional study
Source: Front Public Health. 2024 Oct 16;12:1403634. doi: 10.3389/fpubh.2024.1403634 (PMC11528711; doi:10.3389/fpubh.2024.1403634)
Supplement: Supplementary file 1 [file Table_1.DOCX]

**Section 1 of 4**

**Parent Survey on the HPV Vaccine, the Human Papillomavirus Causing Cervical Cancer**

While filling out the survey, you will come across three different concepts: virus, cancer, and vaccine. It is important to distinguish between them. Their designations are as follows:

1. HPV Virus.

2. Cervical Cancer.

3. The vaccine against the virus.

Your responses will help us measure the knowledge of parents in Saudi Arabia, especially after the inclusion of the HPV vaccine in the vaccination schedule for girls aged 11 and 12.

Your participation in the survey will take approximately 5 minutes.

The data and information collected from this study will be confidential and used only for scientific and research purposes.

**Do you agree to participate in this study?**

Yes

No

---

**Section 2 of 4**

**General Information**

**Gender:**

(1) Male

(0) Female

**Your age in Gregorian calendar:**

**Year of birth:**

**I am employed in the:**

(1) Public/Government sector

(0) Private/International sector

(1-) Unemployed

**My profession is related to the:**

(1) Educational field

(2) Health field

(1-) Not related

**Years of experience:**

**Highest educational qualification:**

(1) Diploma

(2) Bachelor's degree

(3) Master's degree

(4) Doctorate

**I read about medical issues regularly:**

(4) Yes, to a great extent

(3) Yes, to a moderate extent

(2) Yes, to a small extent

(0) I never read

**Marital status:**

(1) Single

(2) Married

(3) Other

**Number of children:**

(0) 0

(1) 1

(2) 2

(3) 3

(4) 4 or more

(1-) Not applicable

**Number of daughters:**

(0) 0

(1) 1

(2) 2

(3) 3

(4) 4 or more

(1-) Not applicable

**Number of daughters aged 10-18 years:**

(0) 0

(1) 1

(2) 2

(3) 3

(4) 4 or more

(1-) Not applicable

**I have been diagnosed with cervical cancer:**

(0) No

(1) Yes

(1-) Not applicable

**I know or have heard of a woman or girl diagnosed with cervical cancer:**

(0) No

(1) Yes

**I have heard about the cervical cancer vaccine through:** (Select all that apply)

(0) No

(1) Yes

1. Awareness from my workplace

2. When I was at university

3. Visual, auditory, or written media

4. Reading research/attending conferences

5. Friends/acquaintances/family

6. I have never heard of this topic before

---

**Section 3 of 4**

**Assessing Your Knowledge of Cervical Cancer, HPV Virus, and the Vaccine**

**Knowledge about Cervical Cancer**

(2) True

(1) False

(0) I don’t know

1. The main cause of all types of cervical cancer is the human papillomavirus (HPV).

2. Symptoms of cervical cancer include pain during intercourse and vaginal bleeding after intercourse.

3. Cervical cancer is diagnosed by taking a cervical smear.

4. Cervical cancer can be treated and completely cured if detected early.

5. There are 5 stages in the development of cervical cancer.

**Knowledge about HPV Virus**

(2) True

(1) False

(0) I don’t know

1. HPV infection has clear symptoms on the female reproductive system.

2. There is a specific PCR test to detect HPV.

3. There is currently a treatment to eradicate HPV.

4. The main mode of transmission of HPV is sexual contact.

5. Men can be infected with HPV just as women can.

6. There are strains of the HPV virus.

**Knowledge about the HPV Vaccine**

(2) True

(1) False

(0) I don’t know

1. The Saudi Ministry of Health recommends receiving the HPV vaccine.

2. The HPV vaccine is given to girls aged 9 to 13 years.

3. The HPV vaccine prevents 70% of cervical cancer cases.

4. The HPV vaccine is administered in two doses, 6-12 months apart.

5. The HPV vaccine is given via intramuscular injection.

---

**Section 4 of 4**

**Measuring Parents' Opinions and Decisions About the Vaccine**

To what extent do you agree with the following statements:

(5) Strongly agree

(4) Agree

(3) Not sure

(2) Disagree

(1) Strongly disagree

1. I believe the HPV vaccine is effective.

2. I believe the HPV vaccine is safe.

3. I should avoid talking to my daughters about sexual education.

4. I should encourage my daughters to get the vaccine.

5. Awareness campaigns about the importance of the HPV vaccine will encourage vaccination.

6. Girls should be allowed to receive the HPV vaccine without parental consent.

7. A "sexual education" course should be included in the school curriculum.

8. I believe that a religious fatwa has a significant impact on encouraging vaccination.

9. I believe that expert medical recommendations have a significant impact on encouraging vaccination.

**As a parent, how would the following factors influence your encouragement of your daughter to receive the HPV vaccine:**

To what extent do you agree with the following statements:

(5) Very positive impact

(4) Positive impact

(3) No impact

(2) Negative impact

(1) Very negative impact

1. A recommendation from a doctor will influence me.

2. Advice from relatives and friends will influence me.

3. My readings about HPV and its vaccine will influence me.

4. Information that HPV is not a global pandemic will influence me.

5. Information that HPV transmission is often linked to sexual activity will influence me.

6. Information that the HPV vaccine is not mandatory will influence me.

7. Government guidelines to receive the vaccine will influence me.

8. My personal convictions about vaccines will influence me.

9. The likelihood of my daughter contracting HPV will influence me.

10. The likelihood of my daughter developing cervical cancer will influence me.

11. The likelihood of side effects from the vaccines will influence me.

12. The prevalence of cervical cancer will influence me.

13. The availability of the HPV vaccine for free will influence me.

To what extent do you agree with the following statements:

(5) Strongly agree

(4) Agree

(3) Not sure

(2) Disagree

(1) Strongly disagree

1. My experience with the COVID-19 vaccine encourages me to advocate for the HPV vaccine.

2. I recommend receiving the HPV vaccine for the necessary protection against cervical cancer.

3. My recommendation to receive the HPV vaccine does not mean I am convinced to give it to my family.

4. I will not advocate for the HPV vaccine if religious scholars object to it.

5. I will not advocate for the HPV vaccine if it is found to have side effects.

6. I will not advocate for the HPV vaccine if it leads to moral decay and the adoption of sexual freedom concepts.

**Finally, we thank you for your cooperation and provide you with this space to write any comments you would like to mention about the survey.**
