# Supplementary material for: Factors influencing human papillomavirus vaccine uptake among parents and teachers of schoolgirls in Saudi Arabia: a cross-sectional study
Source: Front Public Health. 2024 Oct 16;12:1403634. doi: 10.3389/fpubh.2024.1403634 (PMC11528711; doi:10.3389/fpubh.2024.1403634)
Supplement: Supplementary file 2 [file Table_2.DOCX]

**Teachers**

**Section 1 of 4**

**Survey for Teachers on the Human Papillomavirus (HPV) Vaccine That Causes Cervical Cancer**

While filling out the survey, you will encounter three different concepts: virus, cancer, and vaccine. You need to distinguish between them, and you will find the following terms:

1. HPV Virus.

2. Cervical Cancer.

3. The vaccine against the virus.

Your answers will help us measure the knowledge of teachers in Saudi Arabia, especially after the inclusion of the HPV vaccine in the vaccination schedule for girls aged 11 and 12 years.

Participating in the survey will take approximately 5 minutes.

The data and information collected from this study will be confidential and will only be used for scientific and research purposes.

**I agree to participate in this study**

Yes

No

---

**Section 2 of 4**

**General Information**

The year you were born:

I work in an educational sector:

(1) Public/Governmental

(0) Private/International/Other

My role at the school is:

(1) Educational

(2) Administrative

(3) Health-related

Years of experience:

Highest academic qualification:

(1) Diploma

(2) Bachelor's Degree

(3) Master's Degree

(4) Doctorate

I regularly read about medical matters:

(4) Yes, to a large extent

(3) Yes, to a moderate extent

(2) Yes, to a small extent

(0) I never read

Marital status:

(1) Single

(2) Married

(3) Other

Number of children:

(0) 0

(1) 1

(2) 2

(3) 3

(4) 4 or more

(1-) Not applicable

Number of daughters:

(0) 0

(1) 1

(2) 2

(3) 3

(4) 4 or more

(1-) Not applicable

Number of daughters aged 10-18 years:

(0) 0

(1) 1

(2) 2

(3) 3

(4) 4 or more

(1-) Not applicable

I have been diagnosed with cervical cancer:

(0) No

(1) Yes

(1-) Not applicable

I know or have heard of a woman or girl diagnosed with cervical cancer:

(0) No

(1) Yes

I have heard about the cervical cancer vaccine through: (select all that apply)

(0) No (1) Yes

1. Awareness from school

2. During my university years

3. Visual, audio, or print media

4. Reading research/attending conferences

5. Friends/Acquaintances/Family

6. I have never heard of this topic before

---

**Section 3 of 4**

**Measuring Your Knowledge of Cervical Cancer, HPV, and the Vaccine**

**Knowledge about Cervical Cancer**

(2) True

(1) False

(0) I don't know

1. The main cause of all types of cervical cancer is the HPV virus.

2. Symptoms of cervical cancer include pain during intercourse and vaginal bleeding after intercourse.

3. Cervical cancer is diagnosed by taking a Pap smear.

4. Cervical cancer can be treated and completely cured if detected early.

5. There are 5 stages of cervical cancer development.

**Knowledge about the HPV Virus**

(2) True

(1) False

(0) I don't know

1. HPV infection has clear symptoms on the female reproductive system.

2. There is a specific PCR test to detect HPV.

3. There is currently a treatment to eliminate HPV.

4. The main mode of transmission for HPV is sexual contact.

5. Men can contract HPV just as women can.

6. There are different strains of HPV.

**Knowledge about the HPV Vaccine**

(2) True

(1) False

(0) I don't know

1. The Saudi Ministry of Health recommends receiving the HPV vaccine.

2. The HPV vaccine is given to girls aged 9 to 13 years.

3. The HPV vaccine prevents 70% of cervical cancer cases.

4. The HPV vaccine is administered in two doses, 6-12 months apart.

5. The HPV vaccine is given as an intramuscular injection.

---

**Section 4 of 4**

**Measuring Teachers' and Mothers' Opinions and Decisions about the Vaccine**

To what extent do you agree with the following statements:

(5) Strongly agree

(4) Agree

(3) Not sure

(2) Disagree

(1) Strongly disagree

1. I believe the HPV vaccine is effective.

2. I believe the HPV vaccine is safe.

3. I should avoid discussing sexual education with my students.

4. I should encourage my students to get the vaccine.

5. Conducting awareness campaigns about the importance of the HPV vaccine will encourage vaccination.

6. Girls should be able to get the HPV vaccine without parental consent.

7. Sexual education should be included in the school curriculum.

8. I believe religious fatwas have a significant impact on encouraging vaccination.

9. I believe medical recommendations from experts have a significant impact on encouraging vaccination.

**As a teacher, how will the following factors affect your encouragement of students to receive the HPV vaccine?**

To what extent do you agree with the following statements:

(5) Very positive effect

(4) Positive effect

(3) No effect

(2) Negative effect

(1) Very negative effect

1. A recommendation from a doctor will influence me.

2. Advice from relatives and friends will influence me.

3. My readings about HPV and its vaccine will influence me.

4. The information that HPV is not a global pandemic will influence me.

5. The information that HPV transmission is mainly related to sexual activity will influence me.

6. The information that the HPV vaccine is not mandatory will influence me.

7. Government directives to receive the vaccine will influence me.

8. My personal beliefs about vaccines will influence me.

9. The likelihood of my students contracting HPV will influence me.

10. The likelihood of my students developing cervical cancer will influence me.

11. The likelihood of side effects from vaccines will influence me.

12. The likelihood of the spread of cervical cancer will influence me.

13. The availability of the HPV vaccine for free will influence me.

To what extent do you agree with the following statements:

(5) Strongly agree

(4) Agree

(3) Not sure

(2) Disagree

(1) Strongly disagree

1. My experience with the COVID-19 vaccine encourages me to advocate for the HPV vaccine.

2. I recommend receiving the HPV vaccine for adequate protection against cervical cancer.

3. My recommendation to receive the HPV vaccine does not imply that I am convinced of giving it to my own family.

4. I will not advocate for the HPV vaccine if religious scholars object to it.

5. I will not advocate for the HPV vaccine if it is found to have side effects.

6. I will not advocate for the HPV vaccine if it is found to lead to social decay and the spread of sexual freedom concepts.

Finally, we thank you for your cooperation and provide you with this space to write any comments you would like to mention about the survey.
